# Effect of mass variation on vibration properties of the tooth in drilling operation

**DOI:** 10.1038/s41598-022-05824-5

**Published:** 2022-02-01

**Authors:** Sanja Vujkov, Livija Cveticanin

**Affiliations:** 1 University of Novi Sad, Faculty of Medicine, Department of Dentistry, Hajduk Veljka 6, Novi Sad, Serbia; 2 University of Novi Sad, Faculty of Technical Sciences, Trg D. Obradovica 6, Novi Sad, Serbia; 3 Obuda University, Doctoral School of Safety and Security Sciences, Nepszinhaz u. 8, Budapest, Hungary

**Keywords:** Signs and symptoms, Mathematics and computing, Physics

## Abstract

Dental cavity represent one of the widespread illness of the tooth. Method for treating of the tooth is to drill the cavity and to fill the hole with suitable material. Measurements show that during drilling the tooth vibrates with increasing mass that causes unpleasant feeling for patient. The aim of the paper is to give the theoretical explanation for this phenomena and to give suggestion for vibration elimination. During drilling, mass of the tooth is decreasing and the so called ‘reactive force’ occurs. Drilling and reactive force cause tooth vibration. The system is modeled as a nonlinear time variable system. An analytical procedure for solving of the equation of vibration is developed. The solution is assumed in the form of the generalized trigonometric function with time variable amplitude and phase. It is obtained that not only the amplitude but also the frequency of tooth vibration in drilling are increased. In addition to reactive force the drilling velocity, diameter of the drill tool and spindle speed affect the vibration level. The appropriate values of these parameters would eliminate or decrease the patient bad feeling.

## Introduction

**Figure 1:**
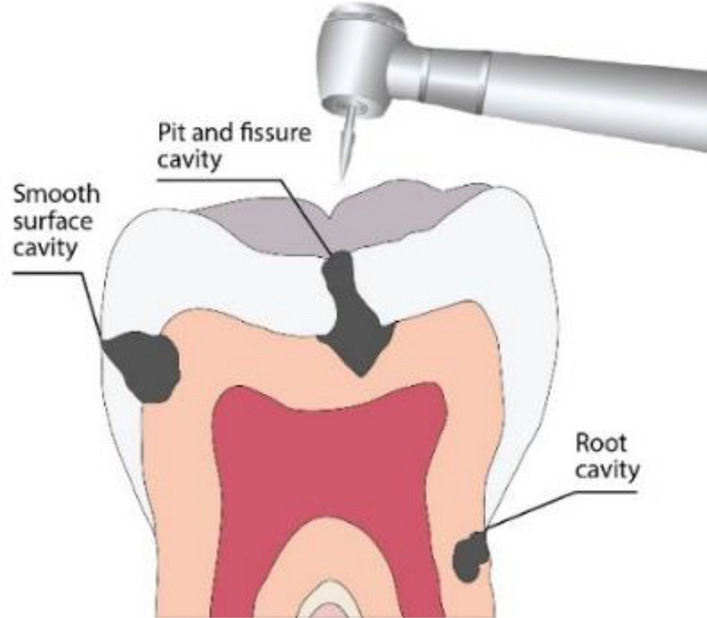
Types of tooth cavities and the drilling direction.

Tooth decay is a damage to a tooth caused by bacteria in mouth which make acids that attack the enamel of the tooth. Tooth decay can lead to cavities (dental caries), which are holes in teeth^1^. Three types of cavities are known: 1. smooth surface cavity (on the smooth side of the tooth), 2. root cavity developed on the surface over the root and 3. pit and fissure cavities which occur on the chewing surface of the tooth (Fig. 1). If tooth decay is not treated, it can cause pain, infection, and even tooth loss. One of the methods of treating the tooth is to remove the decayed tooth tissue by drilling the cavity and to fill the hole with suitable material. Drilling of human teeth in vivo dates form 7500–9000 years ago. In a Neolithic grave yard in Pakistan drilled molar crowns are found^2^. This findings provide evidence for a long tradition of a type of proto dentistry in an early farming culture^2^. Since that time a significant number of improvement in tooth drilling and treating is developed (see for example:^3–5^). In^1^ the review on the repair mechanisms in dental caries are considered. It is shown that the quality of drilling process has a significant influence on the success of healing the tooth. Depending on the type of caries very precise parameters for drilling system are given^6^. For various values of drill diameter the feed rates and the cutting velocity are defined. Diameter of the hole and feed rate determine the drilling force. Distribution of the measured drilling force along the cutting radius is presented in^7^. It is concluded that the force directly affect the stability of the operation and the quality of machined hole. To obtain the required force appropriate dental instruments are suggested, for which it was declared to be keys for the safe and effective removal of dental hard tissues and caries^6^.

In spite of complex investigation, it is evident that some troubles with drilling already exist^8^: there is an unknown temperature distribution and roughness of the surface of the hole^9^, but also vibrations and unpleasant sound and feeling. Using the results obtained in drilling of machines parts, it is concluded that the roughness of the surface is connected with the vibration property^10^. Vibration which occurs during drilling has negative effect on the dentist and also patient. Routine exposure of dentist to consistent vibration during tooth treatment by drilling has ill physical, mental and psychological effects^11^. Vibration and sound gives an unpleasant feeling to patient. Because of that investigation in vibration during drilling are done. Using the non-contact laser vibrometer the vibration displacement amplitudes of high-speed dental hand pieces under loaded are measured. It is obtained that increase of the load corresponds with an increase of vibration amplitude and the maximal vibration amplitude was less than 4 μm^12^. However, in the paper it was concluded that consistent patterns of vibration due to variation in amplitude displacement were not possible to be given. Some additional measurements made on sound, transmitted form tooth to the ear bones, show the increase of frequency during drilling of the tooth^13^. In addition, it is measured that the amplitude of sound increases in time during drilling^14^. However, the causes of this phenomena were not analyzed.

The aim of this paper is to investigate the vibration property of the tooth during drilling. The special attention is given to mass variation during tooth treatment and its effect on amplitude and frequency of vibration. Namely, the vibration is caused with the drilling force and also with the reactive force^15^ which occurs due to tooth mass variation during drilling time and is the product of velocity and the time derivative of mass. Model of the tooth in drilling is assumed as a one degree of freedom slow time variable mass with constant external excitation. Vibration is described with a single second order nonlinear differential equation with time variable parameters. Based on the procedure for solving homogenous strong nonlinear differential equation^16^ with constant parameters a new method for solving non-homogenous equation with time variable parameter is developed. In the solution the generalized trigonometric function (GTF)^17^ is introduced. Approximate solution gives the frequency and amplitude variation in time due to mass variation. In the paper the procedure is applied for human tooth during drilling on the pit and fissure cavities. Analytical result is compared with numerical. Research in influence of the reactive force on vibration properties is done, and the result is compared with that where this force is omitted. The theoretical consideration is compared with experimentally obtained results which are already published (see^14,18^).

## Method

In this section the vibration of the tooth during drilling operation is analytically considered. The tooth system is modeled as a forced oscillator with time variable parameter. Motion of the oscillator is also mathematically modeled. The approximate procedure for solving of the differential equation is developed. The method is innovative and give the result appropriate for further consideration.

### Model of tooth drilling

**Figure 2:**
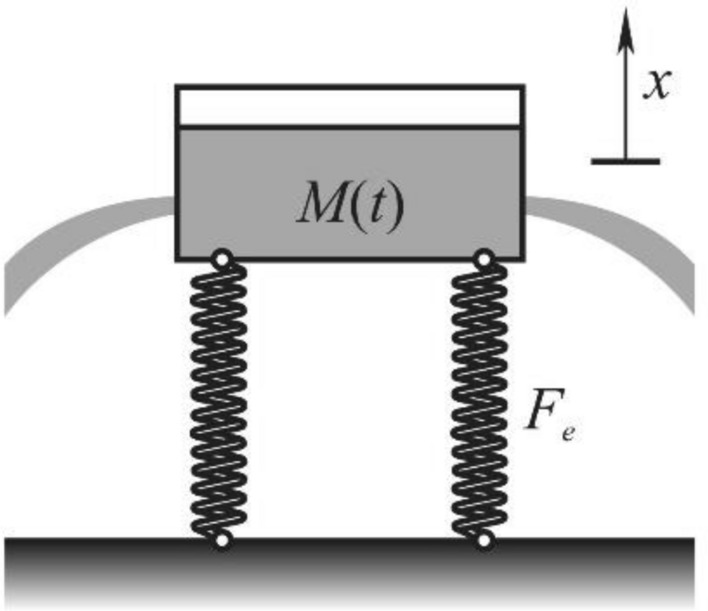
Model of the tooth in drilling.

The model is assumed as a one mass-spring oscillatory system (Fig. 2) with time variable mass *M*(*t*) and spring with nonlinear property. Experimental investigation done on the tooth show that the elastic force in the tooth in axial direction *x* is a strong nonlinear deflection function^19^

1 $$F_{e} = k_{\alpha }^{2} u\left| u \right|^{\alpha - 1}$$

where $k_{\alpha }^{2}$ is the coefficient of rigidity and α is the order of nonlinearity. The parameter α is for the tooth a non-integer which is for the real tooth in the interval (1, 1.1). Mass of the oscillator is decreasing in time. The mass function is $M\left( t \right) = m_{0} - \frac{{\rho D^{2} \pi nf_{r} t}}{4}$ where D, *n* and *f*_*r*_ are parameters of the drilling tool and $\rho$ and *m*_0_ are tooth density and mass parameters, respectively. Comparing the values in mass function it is seen that the feed rate *f*_*r*_ is a small parameter, i.e. *f*_*r*_ = $\varepsilon < < 1,$ and that the mass variation is slow. Introducing the ‘slow time’ $\tau = \varepsilon t$ the mass variation is obtained to be a slow function

2 $$M\left( \tau \right) = m_{0} - \frac{{\rho D^{2} \pi n}}{4}\tau$$

Mass variation produces the reactive force, often called Meshchersky force^15^, which depends on mass variation rate and velocity. (It is worth to be said that this type of forces exist only in the case of mass variation in time.) Using the mathematical relation $\frac{dM}{{dt}} = \varepsilon \frac{dM}{{d\tau }}$ the reactive force follows as

3 $$F_{r} = \varepsilon \frac{dM\left( \tau \right)}{{d\tau }}\dot{u}$$

The reactive force is varying in time.

On the tooth the drilling force $F_{f}$ in the axial direction along the longitudinal axis of the cavity (see Fig. 1) acts. During the drill entrance the drilling force increases rapidly and then decreases to a stable constant magnitude^7^. The force is usually considered to be the linear product of diameter of the drilling tool, feed ratio and a coefficient $K_{f}$ which depends of the properties of the tooth^7^: $F_{f} = K_{f} Df_{r}$. Using the previously mentioned notation for the small parameter (*f*_*r*_ = ε), the drilling force is modelled as

4 $$F_{f} = \varepsilon F$$

where $F = K_{f} D$. In this paper the steady state motion with the constant drilling force (4) is considered. The transient motion as a short time motion is not of special interest for the matter.

### Mathematical model

Using (1), (3) and (4) mathematical model of the tooth vibration in axial direction (see Fig. 2) due to mass variation and axial forces is

5 $$m\left( \tau \right)\ddot{u} + ku\left| u \right|^{\alpha - 1} = - \varepsilon \frac{dm\left( \tau \right)}{{d\tau }}\dot{u} + \varepsilon F$$

The relation (5) is a second order nonlinear differential equation with slow time variable mass. The nonlinearity is of integer or non-integer order α. For simplification, let us introduce the new variable $\left| x \right|^{\alpha - 1} = u\left| u \right|^{\alpha - 1} - \frac{\varepsilon F}{k}.$ Substituting *x* into (5) and neglecting the second order small values ${\mathcal{O}}\left( {\varepsilon^{2} } \right)$ we have

6 $$\ddot{x} + K\left( \tau \right)x\left| x \right|^{\alpha } = - \frac{\varepsilon }{m\left( \tau \right)}\frac{dm\left( \tau \right)}{{d\tau }}\dot{x}$$

where $\left( \tau \right) = \frac{k}{m\left( \tau \right)}$ . In this paper the approximate solving procedure for the strong nonlinear equation with time variable parameter (6) is developed.

### Solving procedure for strong nonlinear oscillator with time variable parameter

The procedure suggested in the paper is based on the solution of the strong nonlinear differential equation

7 $$\ddot{x} + Kx\left| x \right|^{\alpha - 1} = 0$$

where $K = \frac{k}{{m_{0} }}$ and *ε* = 0. The idea of the approximate procedure is based on the assumption that Eq. (6) represents the perturbed version of Eq. (7), i.e. of a pure nonlinear oscillator with constant parameter^20^. According to this assumption, it is supposed that the solution of (6) has to be the perturbed version of solution of the strong nonlinear Eq. (7).

The Eq. (7) has the exact solution *x* and the time derivative $\dot{x}$ in the form of the generalized trigonometric function (GTF)^17^, i.e.

8 $$x = Asin_{2,\alpha + 1} \left( {\omega t + \theta } \right),$$

9 $$\dot{x} = A\omega cos_{2,\alpha + 1} \left( {\omega t + \theta } \right),$$

with the frequency of the function $\omega = A^{{\frac{\alpha - 1}{2}}} \sqrt {\frac{2K}{{\alpha + 1}}}$, where $sin_{2,\alpha + 1} \left( {\omega t + \theta } \right)$ and $cos_{2,\alpha + 1} \left( {\omega t + \theta } \right)$ are the sine and cosine GTF, *A* and *θ* are arbitrary values. (It is known that $\frac{d}{dt}sin_{2,\alpha + 1} t = cos_{2,\alpha + 1} t$).

The time perturbed versions of (8) and (9) are

10 $$x = A\left( t \right)sin_{2,\alpha + 1} \psi \left( t \right),$$

11 $$\dot{x} = A\left( t \right)\omega \left( \tau \right)cos_{2,\alpha + 1} \psi \left( t \right)$$

where the amplitude *A* and phase *ψ* are time variable and

$$\dot{\psi }\left( t \right) = \omega \left( \tau \right) + \dot{\theta }\left( t \right),\quad \omega \left( \tau \right) = A\left( t \right)^{{\frac{\alpha - 1}{2}}} \sqrt {\frac{2K\left( \tau \right)}{{\alpha + 1}}}$$

According to the procedure, it is assumed that (10) and (11) are solutions of (6). Comparing the derivative of (10), $\dot{x} = A\omega cos_{2,\alpha + 1} \psi \left( t \right) + Asin_{2,\alpha + 1} \psi + A\dot{\theta }cos_{2,\alpha + 1} \psi$, with the assumed function (11), the following constraint is obtained

12 $$Asin_{2,\alpha + 1} \psi + A\dot{\theta }cos_{2,\alpha + 1} \psi = 0$$

where $A\left( t \right) = A,$ $\psi \left( t \right) = \psi \theta \left( t \right) = \theta.$ Using (10) and the derivative of (11), i.e. $\frac{d}{dt}cos_{2,\alpha + 1} t = - \frac{\alpha + 1}{2}sin_{2,\alpha + 1}^{\alpha } t$, the Eq. (6) transforms into

13 $$\dot{A}\omega cos_{2,\alpha + 1} \psi - A\omega \dot{\theta }sin_{2,\alpha + 1} \psi = \frac{2\varepsilon }{{\left( {\alpha + 1} \right)}}\left( { - \frac{1}{m}\frac{dm}{{d\tau }}A\omega cos_{2,\alpha + 1} \psi - \frac{1}{2}A\omega \frac{1}{K}\frac{dK}{{d\tau }}cos_{2,\alpha + 1} \psi } \right)$$

Some modification of (12) and (13) yield

14 $$\begin{gathered} \dot{A} = \frac{2\varepsilon }{{\left( {\alpha + 1} \right)\omega }}\left( { - \frac{1}{m}\frac{dm}{{d\tau }}A\omega cos_{2,\alpha + 1} \psi - \frac{1}{2}A\omega \frac{1}{K}\frac{dK}{{d\tau }}cos_{2,\alpha + 1} \psi } \right)cos_{2,\alpha + 1} \psi , \hfill \\ \dot{\theta } = - \frac{2\varepsilon }{{\left( {\alpha + 1} \right)A\omega }}\left( { - \frac{1}{m}\frac{dm}{{d\tau }}A\omega cos_{2,\alpha + 1} \psi - \frac{1}{2}A\omega \frac{1}{K}\frac{dK}{{d\tau }}cos_{2,\alpha + 1} } \right)sin_{2,\alpha + 1} \psi ) \hfill \\ \end{gathered}$$

Equations (14) represent the transformed version of (6) into two coupled first order differential equations. To find the solution of (14) is not an easy task. In general, there is not a closed form solution. This is at this point the averaging over the period of GTF is done. Thus, for averaged functions^17^ $\left\langle cos_{2,\alpha + 1} \psi \right\rangle= 0, \left\langle sin_{2,\alpha + 1} \psi \right\rangle= 0, \left\langle cos_{2,\alpha + 1}^{2} \psi \right\rangle= \frac{\alpha + 1}{{\alpha + 3}}, \left\langle sin_{2,\alpha + 1} \psi cos_{2,\alpha + 1} \psi = 0 \right\rangle$ where $\left\langle \cdot \right\rangle = \frac{1}{{2{\Pi }_{\alpha } }}\mathop \smallint \limits_{0}^{{2{\Pi }_{\alpha } }} \left( \cdot \right)d\psi$ and $2{\Pi }_{\alpha } = \frac{4}{\alpha + 1}B\left( {\frac{1}{\alpha + 1},\frac{1}{2}} \right)$ with beta B special function^21^ the averaged equations of vibration follow as

15 $$\dot{A} = - \frac{2\varepsilon A}{{\left( {\alpha + 3} \right)}}\left( {\frac{1}{m}\frac{dm}{{d\tau }} + \frac{1}{2K}\frac{dK}{{d\tau }}} \right),\;\;\;\dot{\theta } = 0$$

Some special cases of vibration would be considered.

## Results

Three special cases of vibration for the case tooth drilling are considered: (1) motion under reactive force, (2) motion when reactive force is omitted, (3) mass variation is neglected.

### Influence of the reactive force

According to (15) the averaged equations of vibration simplify into

16 $$\dot{A} = - \frac{\varepsilon }{{\left( {\alpha + 3} \right)}}\frac{1}{m}\frac{dm}{{d\tau }}A, \dot{\theta } = 0.$$

Integrating (16) and using the initial conditions $A\left(0\right)$ and $\theta \left(0\right)=0,$ we obtain the relations for amplitude, phase and frequency variation

17 $$A = A\left( 0 \right)\left( {\frac{{m_{0} }}{m}} \right)^{{\frac{1}{\alpha + 3}}} , \quad \theta = \theta \left( 0 \right) = 0, \;\;\;\;\omega \left( \tau \right) = A\left( 0 \right)^{{\frac{\alpha - 1}{2}}} \sqrt {\frac{2}{\alpha + 1}} \left( {\frac{{m_{0} }}{m}} \right)^{{\frac{\alpha + 1}{{\alpha + 3}}}} \sqrt {\frac{k}{{m_{0} }}} .$$

The initial amplitude is $\left( 0 \right) = \left( {\frac{\alpha + 1}{2}\frac{{v_{0}^{2} }}{{\frac{k}{{m_{0} }}}}} \right)^{{\frac{1}{\alpha + 1}}}$ , where according to the law of linear momentum, the initial velocity is $v_{0} = \frac{{F_{f} }}{n} = \frac{{\pi DF_{f} }}{v}$. Analyzing (17) it is seen that for higher velocity, the amplitude and frequency of vibration is smaller. In addition, for larger diameter of the drill tool, the amplitude and frequency of vibration is higher. The approximate analytical expression of vibration is

18 $$u = \left( {A\left( 0 \right)^{\alpha } \left( {\frac{{m_{0} }}{m}} \right)^{{\frac{\alpha }{\alpha + 3}}} sin_{2,\alpha + 1} \psi \left| {sin_{2,\alpha + 1} \psi } \right|^{\alpha - 1} + \frac{\varepsilon F}{k}} \right)^{{\frac{1}{\alpha }}} .$$

**Figure 3:**
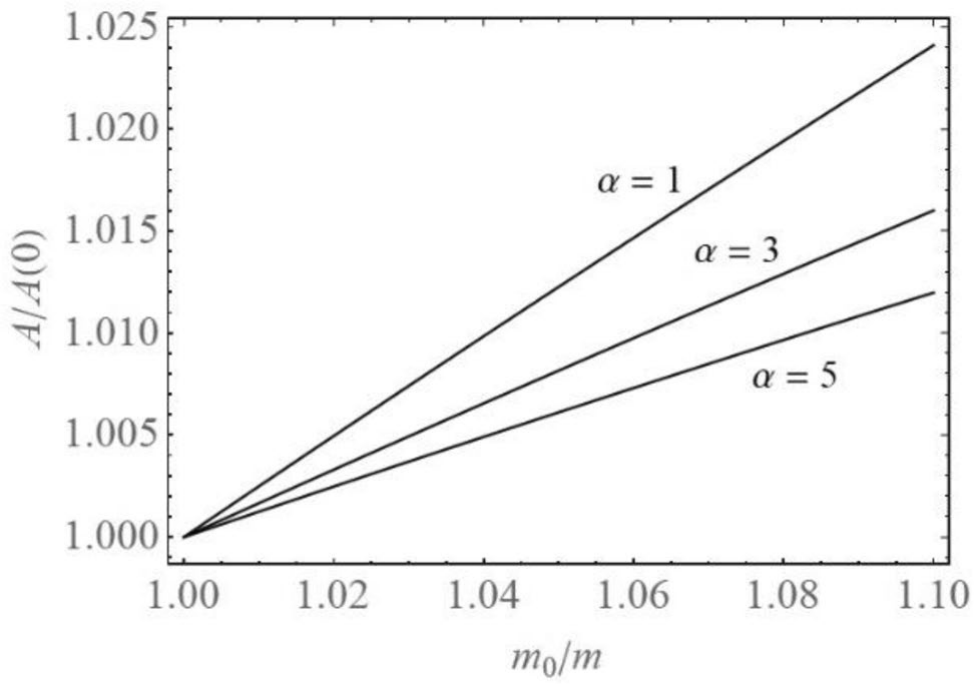
Amplitude-mass diagrams for various α.

It is obvious that the amplitude and frequency of vibration are time dependent. In Fig. 3 the dimensionless amplitude—mass diagrams for various values of $\alpha$ are plotted. It is shown that the amplitude of vibration is increasing with mass increase of the mass ratio *m*_0_/*m*. The amplitude variation does not depend of the coefficient of tooth rigidity, but it depends on the order of nonlinearity. The amplitude increase is faster for lower orders of nonlinearity.

**Figure 4:**
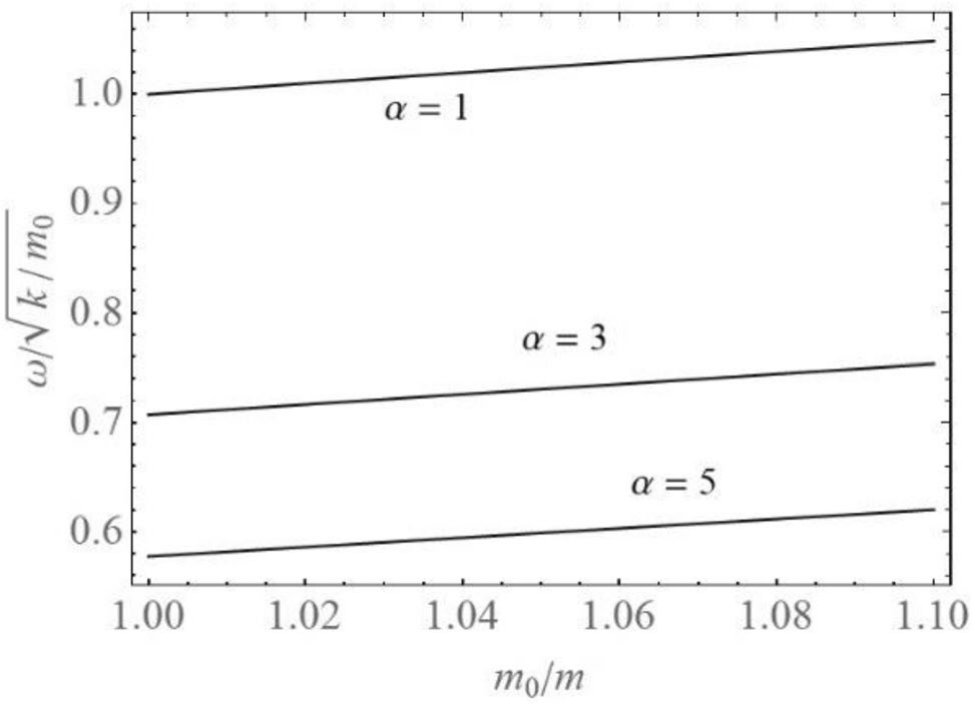
Frequency-mass ratio for various α.

In Fig. 4 the frequency—mass variation diagrams for various values of $\alpha$ are plotted. The frequency of vibration increases with increase of the mass ratio *m*_0_/*m*. The frequency is smaller for higher order of nonlinearity, but the velocity of frequency increase with mass ratio is almost the same for all orders of nonlinearity. In addition, the frequency off vibration depends on the initial amplitude *A*(0).

For the linear case when α = 1 and GTF transforms into the ordinary trigonometric function $si{n}_{\mathrm{2,2}}\psi =sin\psi$ the equation of motion is

19 $$u = A\left( 0 \right)^{{}} \left( {\frac{{m_{0} }}{m}} \right)^{\frac{1}{4}} sin\psi + \frac{\varepsilon F}{k}$$

with frequency of vibration $\omega \left( \tau \right) = \sqrt {\frac{k}{m\left( \tau \right)}}$ which is only dependent on mass variation and independent on initial conditions.

#### Remark

For the constant value of the tooth mass the natural frequency of the tooth is $\omega =\sqrt{\frac{k}{{m}_{0}}}$. It depends on the properties of the tooth: rigidity *k* and mass *m*_0_. It has to be mentioned that this value differs from the frequency of the drill *Ω* = π *n*/30 which is the linear function of the speed of the spindle of the drilling tool. The effect of drilling is the highest if the two values are close.

### Reactive force is omitted

If the reactive force and additional external forces are omitted the averaged vibration Eqs. (15) are simplified into

20 $$\dot{A} = \frac{\varepsilon A}{{\left( {\alpha + 3} \right)}}\left( {\frac{1}{m}\frac{dm}{{d\tau }}} \right), \dot{\theta } = 0$$

Integrating (20) and using the initial conditions $A\left( 0 \right)$ and $\theta \left( 0 \right) = 0,$ we obtain

21 $$A = A\left( 0 \right)\left( {\frac{m}{{m_{0} }}} \right)^{{\frac{1}{\alpha + 3}}} , \theta = \theta \left( 0 \right) = 0. \omega \left( \tau \right) = A\left( 0 \right)^{{\frac{\alpha - 1}{2}}} \sqrt {\frac{2}{\alpha + 1}} \left( {\frac{m}{{m_{0} }}} \right)^{{\frac{\alpha + 1}{{\alpha + 3}}}} \sqrt {\frac{k}{{m_{0} }}}$$

Comparing (17) and (21) it is obvious that the amplitude and frequency tendency for the case with and without reactive force is opposite. The same case is for the frequency of vibration of the body with decreasing mass: for the case when reactive force is neglected the frequency off vibration is also decreasing, and if it the reactive force is included into calculation the frequency is increasing.

### Mass variation is neglected

For that case it is obtained that the vibration is with constant amplitude *A*(0) and constant frequency $\omega = A\left( 0 \right)^{{\frac{\alpha - 1}{2}}} \sqrt {\frac{2k}{{\left( {\alpha + 1} \right)m_{0} }}}$.

The principal question is, which mathematical model for tooth drilling is the most appropriate one. To answer the question comparison of the analytical results with experimentally obtained ones was necessary.

## Discussion

Applying the laser vibrometry the vibration of the tooth during drilling is measured^12^. It is seen that the amplitude and the frequency of vibration are varying in time. In Fig. 6 the experimentally obtained amplitude – time diagram is plotted. It is obtained that during tooth drilling the amplitude is increasing in the interval of 0.090–0.202 μm^19^. Using the aforementioned theoretical consideration, the experimentally obtained results have to be proved analytically.

It is known that the initial mass of the human tooth is between 0.51 g and 2.28 g^19^ and the mass depends on many factors: oldness of the person, type of the tooth, depth of the root etc. The drill diameter is in the interval 1–5 mm, the drilling velocity is in the interval of 500–2000 rpm and the suggested feed rate for tooth drilling is 0.005–0.06 mm/rev^7^. Assuming the numerical data *D* = 2 mm, *f*_*r*_ = 0.05 mm/rev, *m*_0_ = 1.32 g given in^22^, the mass variation is $m = 1.32 - 0.002t$. Based on the measured value of the elastic force in the tooth it is obtain that the order of nonlinearity is *α* = 1.03^23^ and the rigidity coefficient is approximately *k* = 1 g/s^2^. Substituting the mass relation the equation of motion (7) is

22 $$\left( {1.32 - 0.002t} \right)\ddot{u} + u\left| u \right|^{0.03} = 0.002\dot{u} + 0.02$$

with initial conditions $u\left( 0 \right) = u_{0} = 0.02 {\text{mm}}, \dot{u}\left( 0 \right) = v_{0} = 0.5\frac{{{\text{mm}}}}{s},$ where $u_{0}$ is the initial deflection and $v_{0}$ is the initial velocity due to impact between the tooth surface and the drilling tool. Substituting the numerical values, for the initial amplitude $A\left( 0 \right) = 0.58345$ mm, it is

23 $$u \approx 0.58345\left( {\frac{{m_{0} }}{m}} \right)^{{\frac{1}{4.03}}} sin_{2,2.03} \psi + 0.0194$$

with frequency of the function $\omega \left( \tau \right) = 0.98557{\text{m}}^{ - 0.50372}$.

**Figure 5:**
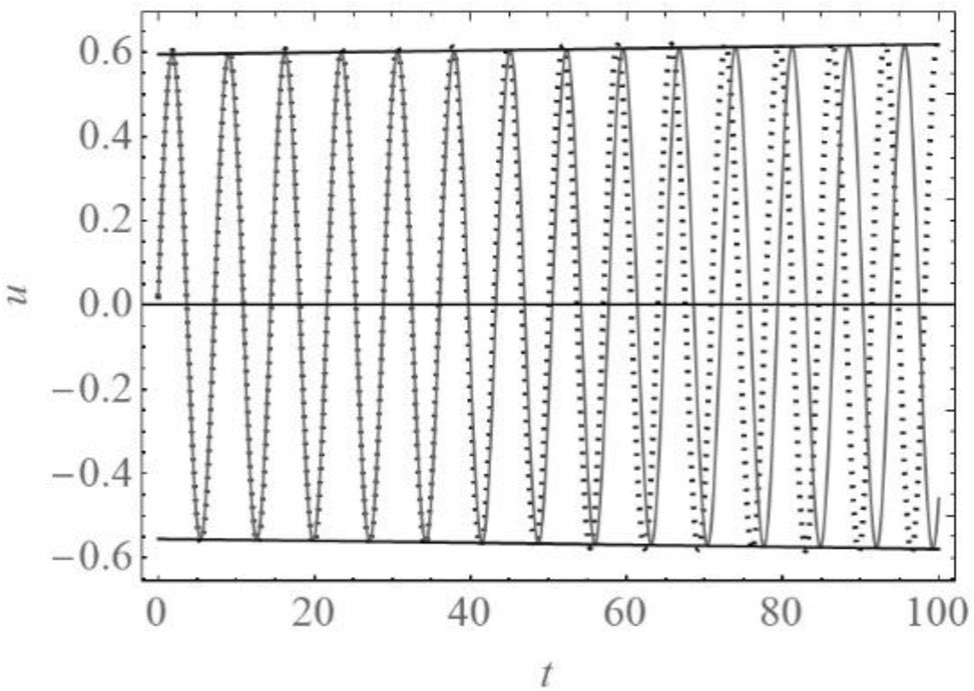
*u-t* diagrams obtained numerically (full line) and analytically (dotted line).

**Figure 6:**
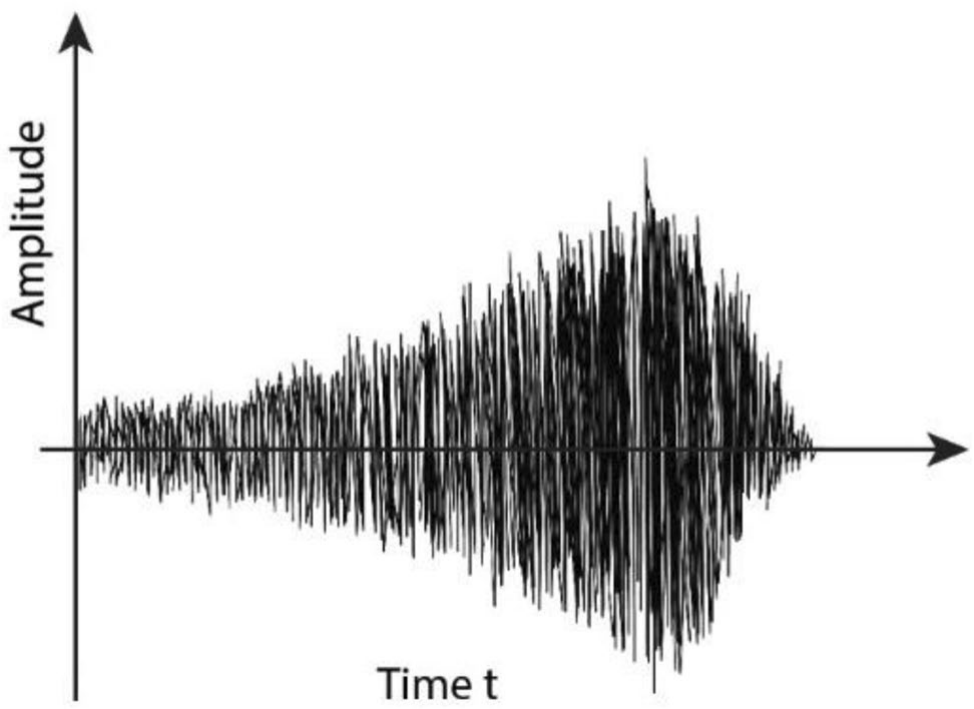
Scheme of the measured amplitude-time diagram.

Applying the Runge Kutta procedure the numerical solution of the Eq. (22) is computed. The solution is compared with the analytical solution (23) and plotted in Fig. 5. It is obvious that the difference between analytical and numerical solution is negligible. Higher differences are evident for the longer time interval. Comparing the solution of (23), shown in Fig. 5, with experimentally obtained amplitude – time diagram^14^ (see Fig. 6) it is obvious the vibration obtained by measuring and that analytically calculated have the same qualitative properties: amplitude of vibration is increasing in time, while the period of vibration is decreasing in drilling of the tooth. Thus, experimentally obtained result is proved with the analytical model and the solving procedure suggested in this paper.

The same result is obtained by measuring of sound in drilling^18^. The frequency and amplitude of the bone conducted sound produced by vibration, recorded and analyzed by the acoustic analysis software, were increasing during tooth drilling. The advantage of the analytic solution is that the influence of parameters which affect the vibration increase can be directly controlled.

## Conclusion

In this paper the influence of the mass variation during the drilling operation on vibration of the tooth is considered. In the consideration the effect of the reactive force is included. The system is modeled as a mass variable system with strong nonlinear property. The nonlinear differential equation with slow time variable parameters is solved. A procedure based on variation of the strong nonlinear differential equation with constant parameter is developed. The solution is assumed in the form of the generalized trigonometric function. The suggested procedure gives results which are in good agreement with numerical and experimental ones. Based on the results it is concluded:

- The amplitude and frequency of vibration of the tooth in drilling are not constant. Both values are increasing in time, while the mass of the tooth is decreasing.
- For higher cutting velocity and spindle speed the increase of the amplitude-time curve is faster than for smaller velocities. If mass decrease is faster, the effect of amplitude of tooth vibration is more significant.
- The effect of drilling depends on the value of the initial tooth mass: for heavier is the tooth, the increase of amplitude of vibration is slower.
- Dimension of the drill-tool affects the vibration. The higher is the diameter of the drill-tool, the amplitude of vibration is higher, and the roughness of the drilled surface increases.
- In theoretical consideration it is shown that neglecting of the reactive force or even omitting the mass variation during drilling of the tooth is not allowed. In spite of the fact that mass variation and the reactive force are quite small in comparison to the total mass of the tooth, their effect is significant.

According to the obtained results it is obvious that unpleasant feeling in the patient is increasing during tooth drilling due to increase of the amplitude and frequency of vibration. To slow down the amplitude and frequency increase the velocity of mass variation has to be decreased. However, it causes prolonging of the drilling process. Therefore, future investigation have to be directed toward optimizing of the drilling parameters with respect to vibration elimination.

## Abbreviations

*D*: Diameter of drilling tool
${f}_{r}$: Feed rate in drilling
*F*_*e*_: Elastic force
*F*_*f*_: Drilling force
*F*_*r*_: Reactive force
*M*: Mass of the tooth
${m}_{0}$: Initial mass of the tooth
$$n$$: Spindle speed
*t*: Time
$$u$$: Displacement
$$\dot{u}$$: Velocity
$$\ddot{u}$$: Acceleration
$\rho$: Density of tooth material
$$\tau$$: Slow time
$$\varepsilon$$: Small parameter
